# Immunossupressed patients admitted into intensive care with infection: risk factors for infection by multidrug resistant pathogens and hospital mortality

**DOI:** 10.1186/2197-425X-3-S1-A125

**Published:** 2015-10-01

**Authors:** M Monteiro, P Medeiros, T Cardoso, G Campello

**Affiliations:** Intensive Care Unit, Hospital de Santo António, Centro Hospitalar do Porto, Oporto, Portugal

## Introduction

Immunosuppression (IS) has been described as a factor associated with mortality in intensive care unit (ICU) and with increased susceptibility to infection by multidrug-resistant pathogens (MDR). Variables that increase these risks have not been completely determined.

## Objectives

Characterization of the population under immunosuppressive therapy (IST) admitted in ICU with infection, and description of risk factors for infection by a MDR pathogen and hospital mortality.

## Methods

Retrospective cohort study of all patients under IST (corticosteroids, chemotherapy or other immunosuppressive drugs) admitted with infection at a 11-bed mixed ICU, in a tertiary care, university-affiliated hospital, between January 2011 and December 2014. Patients who started IS in the same episode were excluded. MDR pathogen was defined as a pathogen resistant to at least three antibiotics from different groups recommended for its treatment. Heavy IST was defined as prednisolone> 20 mg/day and/or multiple IS and/or chemotherapy. The association of several variables with infection by MDR pathogen and with hospital mortality was studied through logistic regression.

## Results

During the study period 91 patients under IST were admitted in the ICU, of those 61 were hospitalized for infection (67%). The mean ± SD age was 59 ± 15 years, 54% were male, and the mean ± SD SAPS II was 52 ± 22 and SOFA 9 ± 4. The underlying diseases identified were: autoimmune diseases (31%), solid tumors (21%), transplanted patients (25%), hematologic diseases (16%) and others (7%); 46 (75%) patients were under heavy IST. The most prevalent foci infection were respiratory (61%) and abdominal (13%). Microbiological documentation of infection was possible in38 patients (62%): 16 (44%) gram negative, 7 (18%) gram positive, 2 (5%) fungus, 2 (5%) other and 11(29%) polimicrobial. MDR pathogens were identified in 8 (21%). Diabetes was significantly associated with infection by a MDR pathogen [OR(95%CI) = 6.5(1.14-37.05),p = 0.035]. Empiric antibiotherapy was adequate in only 34% of the patients. Those infected with MDR pathogen had a significantly higher rate of inadequate antibiotic therapy (75% vs 25%,p = 0.002). Hospital mortality rate was 54%. Variables associated with increased hospital mortality were: male gender [OR(95%CI) = 4.14(1.42-12.09)], chemotherapy [OR(95%CI) = 4.33(1.33-14.14)], duration of treatment < 1 month [OR(95%CI) = 0.19(0.05-0.77)], acute hematological dysfunction [OR(95%CI) = 9.2(2.75-30.79)], acute renal dysfunction [OR(95%CI) = 3.09(1.08-8.82)], total SOFA score [OR per point (95%CI) = 1.169(1.008-1.356)] and SAPS II score [OR per point (95%CI) = 1.030(1.003-1.057)].

## Conclusions

Infection was the most common cause of ICU admission in IS patients. Diabetic had higher prevalence of infection by MDR pathogens. Surprisingly, infections by MDR pathogens or inadequate initial antibiotic therapy were not among the risk factors associated with increased hospital mortality.

